# Effect of silver nanoparticles on tropane alkaloid production of transgenic hairy root cultures of *Hyoscyamus muticus* L. and their antimicrobial activity

**DOI:** 10.1038/s41598-023-36198-x

**Published:** 2023-06-27

**Authors:** Aisha M. Abdelkawy, Shifaa O. Alshammari, Hebat-Allah A. Hussein, Inas M. M. Abou El-Enain, Eman S. Abdelkhalek, Asmaa M. Radwan, Sahar K. M. Kenawy, Doaa A. M. Maaty, Nermine N. Abed, Shadia Sabry, Abeer Mohsen

**Affiliations:** 1 Botany and Microbiology Department, Faculty of Science (Girls Branch), Al-Azhar University, Cairo, Egypt; 2 Biology Department, College of Science, University of Hafr Al-Batin, 31991 Hafr Al-Batin, Saudi Arabia; 3 Biology Department, University College of Nairiyah, University of Hafr Al-Batin, 31991 Nairiyah, Saudi Arabia; 4 Biology Department, Al Darp University College, Jazan University, Gazan, Saudi Arabia

**Keywords:** Biotechnology, Microbiology

## Abstract

The utilization of nanotechnology and biotechnology for enhancing the synthesis of plant bioactive chemicals is becoming increasingly common. The hairy root culture technique can be used to increase secondary metabolites such as tropane alkaloids. *Agrobacterium* was used to induce hairy roots from various explants of *Hyoscyamus muticus*. The effect of nano-silver particles (AgNPs) at concentrations of 0, 25, 50, 100, and 200 mg/L on tropane alkaloids synthesis, particularly hyoscyamine and scopolamine, was studied in transgenic hairy root cultures. Different types of explants obtained from 10-day-old seedlings of *H. muticus* were inoculated with two strains of *Agrobacterium rhizogenes* (15,834 and A4). The antimicrobial activity of an ethanolic extract of AgNPs-induced hairy root cultures of *H. muticus* was tested. The frequency of hairy roots was higher in hypocotyl, root, leaf, and stem explants treated with *A. rhizogenes* strain A4 compared to those treated with strain 15,834. In transgenic hairy root cultures, AgNPs application at a concentration of 100 mg/L resulted in the highest total tropane alkaloid production, which exhibited broad-spectrum antimicrobial activity. The study demonstrated the potential of nano-silver as an elicitor for promoting the production of target alkaloids in *Hyoscyamus muticus* hairy root cultures, which exhibit high biological activity.

## Introduction

Medicinal plants have been the primary source of life-saving drugs for people around the world since ancient times^1^. In addition, plant-based medications are essential sources of several pharmaceuticals^2^. *Hyoscyamus muticus* L. (*H. muticus*), an essential medicinal herb belonging to the Solanaceae family^3^. Scopolamine and hyoscyamine are the most often used chemicals obtained from *Hyoscyamus* species in traditional medicine. However, due to their complex chemical properties, commercial production of these chemicals is challenging and expensive. Therefore, there is an urgent need to use biotechnology techniques to increase these chemicals in the root cells and aerial parts of the plant. Scopolamine and hyoscyamine are widely used to alleviate spasms and cholinergic symptoms in the parasympathetic nervous system^4–6^**.**They are also used as narcotic analgesics to treat Parkinson's disease, asthma, and motion sickness^7^.

According to Zhao et al.^8^, the production of hyoscyamine and scopolamine begins when ornithine converts into putrescine by the enzyme ornithine decarboxylase (ODC). Moreover, tropane alkaloids may be produced at the highest and quickest rate from transgenic root cultures using *Agrobacterium rhizogenes* (RI)^9^. To speed up the accumulation of secondary metabolites in medicinal plants, various methods have been developed, such as high cell product selection, nutrient culture media conditioning, precursor supply, bioreactor system scaling up, hairy root transplantation implementation, plant cell immobilization, and biotransformation^10,11^.

Tissue culture and genetic modification can be used to increase the production of pharmaceuticals. These techniques make it simpler to extract specific materials from plant tissues rather than from the entire plant. The use of genetic modification can also help to control the process of alkaloid biosynthesis, which is important for the development of medicinal compounds^12^.

Plant roots may develop hairy roots from the *RI* (T-DNA) plasmid of *RI Agrobacterium*. The generated hairy roots are more beneficial than the mother plants because of their genetic and biochemical stability, rapid growth, hormone auto-feeding, low cost of lateral branching needs, and potential for multi-enzyme biosynthesis. In addition, these generated hairy roots have a shorter generation time^13,14^. Some signaling molecules may serve as catalysts for the induction and production of secondary metabolites in plants^15^.Signaling components can be utilized as elicitors to trigger the production of targeted secondary metabolites in plant cell cultures. This strategy is effective in inducing the synthesis of secondary metabolites throughout the entire plant using in vivo elicitors.

Nanoparticles (NPs) may act as signaling molecules, starting physiologic and metabolic reactions. The fundamental processes for how NPs could act as signaling molecules to start physiological and metabolic reactions are yet unknown^16^. Due to their unique properties, NPs increasingly used in many other fields as nanotechnology spreads across the physiology and biochemistry fields^17^. In addition, this method may supply novel opportunities for study in agriculture and plant sciences. NPs affect seed viability and plant development for potential use in farming. Nanoparticles accelerated germination and plant development in groundnut plants and boosted antioxidant activity^18,19^.

Moreover, NPs affect plant secondary metabolites through reactive nitrogen/oxygen species, gene expression, and signaling pathways metabolites. Higher plants produce surpass 200,000 secondary metabolites that have various biological activities. Secondary metabolites produced by elicitors are employed in nanomedicines. Furthermore, nanoparticle (NP) routes affect plant secondary metabolism and chemical elicitation^20^.

Lu et al.^21^ observed an increase in antioxidant enzyme activities in *Brassica juncea* seedlings when treated with silver nanoparticles. The elicitation strategy can stimulate the synthesis of specialized metabolites in plant cell cultures by activating genes involved in the secondary metabolism pathways of plants. NP roles in the body are still unknown, but their application as elicitors is growing^22^.

Microorganisms vary widely in their resistance and cause infectious in humans^23^. These diseases pose a significant threat to human life and the economy. The usage of antibiotics is restricted because of the resistance of pathogens against many antibiotics. Natural compounds that limit microbial development are thus a specialized research topic^24^. Essential oils and secondary metabolites found in medicinal plants are valuable resources for the pharmaceutical industry^22^. Medicinal plants have wide biological and antimicrobial activities and still play an important role in contemporary medicine.

The goal of this study is to investigate the effect of silver nanoparticles (AgNPs) on the synthesis of scopolamine, hyoscyamine, and total alkaloids in the hairy roots of *Hyoscyamus muticus* L. The study also intends to assess the antibacterial and antifungal capabilities of ethanolic extracts from hairy root cultures against gram-positive and gram-negative bacteria, yeast, and fungus.

## Materials and methods

### In vitro seeds germination

All plant experiments are conducted in accordance with relevant institutional, national, and international guidelines and legislation. The seeds for the *Hyoscyamus muticus* study were provided by the Egyptian Ministry of Agriculture's Department of Medicinal and Aromatic Plant Research. The seeds were ready for planting after being surface sterilized in 70% ethanol for 1 min, 25% commercial Clorox (5.25 percent sodium hypochlorite) with a few drops of Tween 20 for 20 min, and three washing in sterile distilled water. Seeds were cultured in a solid (MS, Murashige, and Skoog) basal medium with 30 g/L sucrose25 to stimulate in vitro germination after sterilization^25^.

### Induction of hairy roots

The strains 15,834 and A4 of *A. rhizogenes* (obtained from the Agriculture Microbiology Department, National Research Centre) were utilized. Each bacterium strain was grown for 2 days in liquid LB medium (Luria Bertani media)^26^ before being incubated at 28 °C on an orbital shaker at 110 rpm in darkness to achieve an OD of 0.6 at 600 nm.

#### Inoculated explants

After 10 days of seed growth, *Agrobacterium rhizogenes* strains 15,834 or A4 were used to inoculate hypocotyl, leaf, stem, and root explants. After 2 days of dark culture in hormone-free MS media having 3% (w/v) sucrose, 7% (w/v) agar, and 0.1 g L^−1^ Myo-inositol, the inoculated explants were transferred to the same medium supplemented with 200 (mg/L) cefotaxime as an antibiotic. Hairy roots were induced and observed 2 weeks later. The hairy roots were grown in MS medium and subcultured every 10 days after antibiotics were withdrawn for the third time. Every 2 weeks, the colonies were transferred to new 250 (mL) Erlenmeyer flasks containing 30 mL of hormone-free liquid MS media (shaken at 120 rpm at 25 °C in the dark).

### Polymerase chain reaction analysis

(See the supplementary file).

### Impact of AgNPs on the accumulation and synthesis of alkaloids

AgNP at various concentrations, including 0, 25, 50, 100, and 200 mg/L, were employed in this experiment. The AgNPs were bought from Sigma (Aldrich) under catalogue number 730807. The silver nanoparticles were dissolved in ethanol with polyvinylpyrrolidone (PVP). Silver nanoparticles are described using transmission electron microscopy (TEM). TEM imaging has a significantly higher resolution than light-based imaging techniques due to the fact technique uses electrons rather than light to illuminate the sample.

According to data on AgNP characterization, sodium citrate serves as a stabilizing agent in silver dispersion NPs with a 40 nm particle size (TEM), 0.02 mg/mL in an aqueous buffer. After filtering the AgNPs solution via a micro-filter to sterilize it, the AgNPs were added to an autoclaved, liquid-free MS medium under sterile circumstances at various quantities. The sugar concentration in the in vitro growth medium was set at 30 g/L. After having its pH adjusted to 5.6, the culture medium was autoclaved for 20 min at 121 °C. Separate pieces of hairy roots were transplanted into the growth chamber and kept to grow for 30 days at a constant temperature of 25 degrees Celsius and absolute darkness. The experimental methodology was designed using a totally randomized approach. After (30) days of in vitro cultivation, the fresh and dried weights per jar (in g/jar) were recorded.

### Hairy roots fresh and dry weights and measurements

The dry weights of hairy roots were measured 3 weeks after AgNPs were applied. The inoculation was performed with (100 mg/jar). After weighing fresh weight, roots were left in the air for 48 h in the dark at room temperature to dry to keep the thermo-sensitive biological components.

### Extraction and determination of tropane alkaloids using HPLC

The alkaloids were extracted using the Kamada et al. technique^27^.To extract tropane alkaloids, hairy root samples (500 mg) were soaked in 10 mL of ethanol containing 2% H_2_SO_4_ for (48 h) to extract tropane alkaloids. Adding 14% ammonia at a pH of 8.0 combined with dried, filtered extracts for alkaloid release with using sodium sulfate to remove the water residue. The alkaloids were then re-extracted three times with ether (3 mL), followed by washing with a 10% KCl solution to remove silver ions and salts. Before using high-performance liquid chromatography (HPLC), the filtrate extracts were dried at room temperature, and 2 mL of phosphate buffer was added to each fraction. The HPLC study was carried out in a water system outfitted with an HPLC pump, a UV detector (dual absorbance) at 204 nm, and a UV-absorbance detector (single absorbance). Each sample (20 μL) was injected onto a reversed-phase HPLC column [RP-18, 250, 4.6 (mm), 5-m particle size, Shimadzu, Japan] with NaH_2_PO_4_ (0.78 g/L) and H_3_PO_4_ (0.2 mL) in water (pH 2.6) as solvent A and acetonitrile as solvent B, with isocratic elution at 40 °C with a ratio of 90%. Using varying quantities of atropine (Sigma Aldrich, USA) as the standard (300, 200, 100, 50, 20, and 10 g/mL) to create a standard calibration curve. The correlation between concentration and peak area of the standard was calculated using the minimum square technique (R2 value).

### Antimicrobial activity

Yeast *Candida albicans* (ATTC90028), fungal strains *Aspergillus terreus* (SQu14026), *Fusarium chlamydosporum* (F25), *Alternaria alternata* (Te19), and *Microsporum canis* (IFM 45,829), and bacterial strains *Staphylococcus aureus* (ATCC 29,213), *Bacillus subtilis* (NBRC:13,719), *Pseudomonas aeruginosa* (ATTC35639), *Escherichia coli* (ATTC11775), and *Klebsiella pneumonia* (ATCC 15,380) were used to evaluate antimicrobial activity of hairy root ethanolic extract by using disc diffusion method^28^. A final concentration of 200 (µg/mL) was attained by diluting the ethanol extract with dimethyl sulfoxide (DMSO). Twenty microliters of extract were applied to sterile discs. Plates of nutrient agar and Czapek's agar media were seeded with 100 µL of bacterial and fungal inoculum (0.5 Mac Farland^29^. The Petri dishes containing bacteria were incubated at 37 degrees Celsius for 24 h, while those containing fungi were incubated at 28 °C for 72 h. DMSO-treated discs were used as the negative control, whereas ampicillin and fluconazole were used as positive controls for bacterial and fungal strains, respectively. Antimicrobial activity was evaluated by measuring the diameter of the inhibitory zones.

### The statistical analysis

The arrangement for each experiment was completely randomized with three replicates. The data was subjected to Analysis of Variance (ANOVA) as described by Snedecor and Cochran^30^ followed by Duncan's Multiple Range Test (DMRT)^31^ at *P* < 0.05 for the significant differences between treatments using COSTAT package ver. 6.4 (CoHort software, Monterey, USA).

## Results

**Figure 1:**
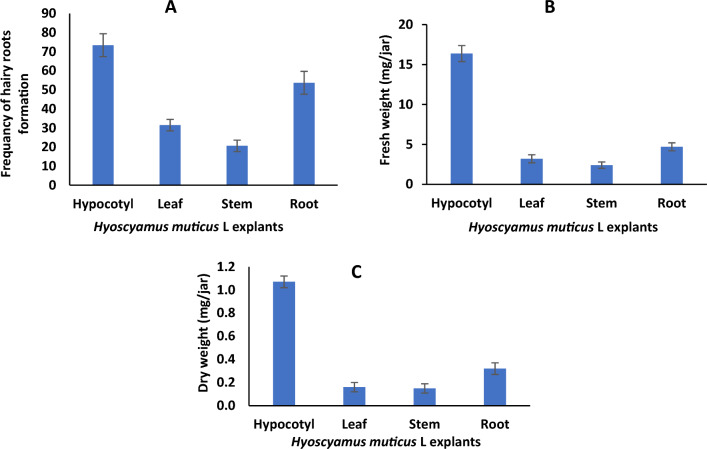
Effect of inoculation *Hyoscyamus muticus* L explants with *Agrobacterium rhizogenesis* strain A4 on frequency, fresh and dry weights (mg/ jar) of hairy roots formation.

**Figure 2:**
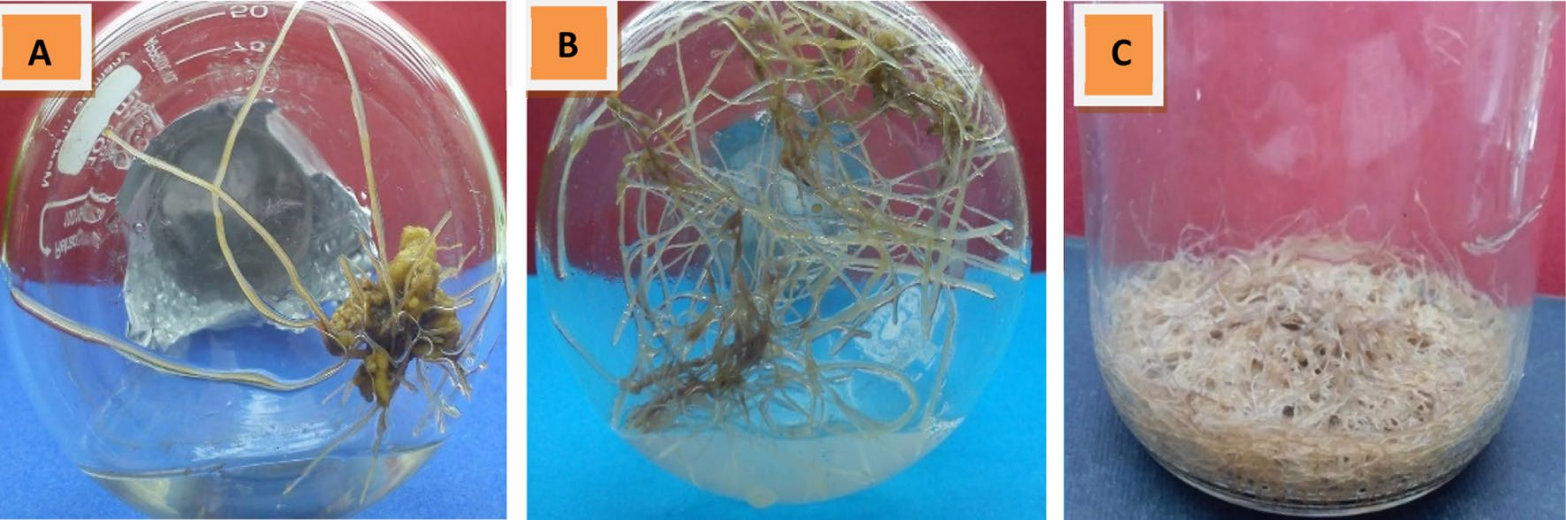
Hairy roots produced from hypocotyl explants of *Hyoscyamus muticus* L inoculated with *Agrobacterium rhizogenesis* strain 15,834 (**A**) and A4 (**B**) after 7 days, and A4 after 30 days (**C**).

The effect of inoculating different *Hyoscyamus muticus* L. explants with two strains of *A. rhizogenes* on the development of hairy roots was investigated. After 10 days of seed culture, the hypocotyl, leaf, stem, and root explants were excised and inoculated with two strains of *Agrobacterium rhizogenes*: 15,834 and A4. After a period of 10 days following inoculation, the frequency percentage of hairy root formation, as well as the fresh and dried weights (measured in mg per jar), were recorded. Data in (Fig. 1) depicts the highest frequencies (percentages) of hairy root formation in hypocotyl, root, leaf, and stem explants treated with *A. rhizogenes* strain A4: 73.4%, 53.7%, and 31.5%, 20.2%, respectively. Fresh weights were 16.37, 4.7, 3.2, and 2.4 (mg/jar), respectively. The dry weights of hypocotyl, root, leaf, and stem explants treated with *A. rhizogenes* strain A4 were 1.07, 0.32, 0.16, and 0.149 mg/jar, respectively (Fig. 2). However, there was no response with *A. rhizogenes* (strain 15,834), which produced tumorigenic calli and a few hairy roots; thus, only the A4 strain would be employed in the subsequent experiment.

### Polymerase chain reaction analysis

The purpose of the PCR analysis was to determine the presence of T-DNA from *Agrobacterium rhizogenesis* (Ri plasmid). The T-DNA at the 580 bp of (Ri) plasmid was integrated and expressed in the hairy root of *H. muticus* infected with the two *A. rhizogenesis* strains utilized (A4 and 15,834) compared to the DNA isolated from normal *H. muticus* roots, where no T-DNA was detected (Supplementary Fig. S1).

### Effects of AgNPs on the development of hairy roots

**Figure 3:**
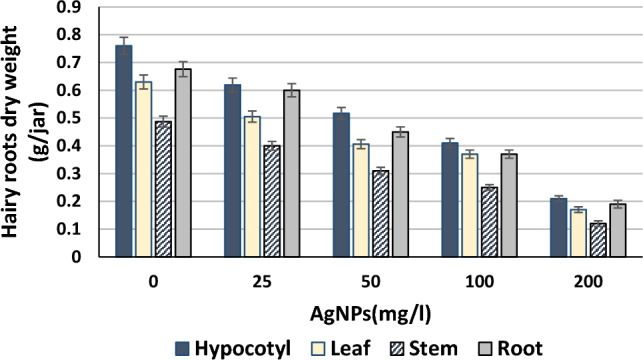
Effect of AgNPs (mg/L) supplemented in nutrient media of hairy roots dry weights of *H. muticus*.

The different concentrations of silver nanoparticles (0, 25, 50, 100, and 200 (mg/L), were applied to transgenic hairy root cultures (100 mg/jar) treated with the *Agrobacterium* strain (A4). The dry weights of hairy root cultures derived from hypocotyl, root, leaf, and stem explants were 0.76, 0.676, 0.63, and 0.487 (g/jar), respectively (Fig. 3). Furthermore, the control had the highest dry weight, followed by treatments containing 25, 50, 100, and 200 (mg/L) of AgNPs for all hairy roots generated from the various explants employed. However, increasing the concentration of AgNPs decreased the growth of hairy roots while increasing the alkaloids content. This indicates that the application of AgNPs acts as an elicitor/stressor, depending on the applied concentration.

### AgNPs treatment affects total alkaloids, hyoscine, and hyoscyamine accumulation and synthesis

Table 1 shows that the highest total alkaloids (3.57, 3.45, 3.43, 3.32, and 3.25%) were recovered from hairy root cultures coming from root explants treated with 100, 50, 200, 25, and 0 mg/L AgNPs, respectively. Root, leaf, stem, and hypocotyl explants recorded a high accumulation rate of total alkaloids, respectively. In addition, a high accumulation rate of hyoscine, 0.037, 0031, 0.029, 0.027, and 0.023% were recorded with hairy root cultures produced from root explants treated with 100, 50, 200, 25, 0 (control) mg/L of AgNPs, respectively. The maximum value of hyoscine responded from hairy root cultures produced from root, leaf, stem, and hypocotyl explants. Moreover, the maximum value of hyoscyamine was 0.046% in the extract of hairy roots derived from root explants exposed to AgNPs at (100 mg/L). The extracted hairy root cultures of root, leaf, stem, and root explants showed the best results of hyoscyamine accumulation, respectively.

**Table 1:** HPLC analysis of total alkaloids, hyoscine, and hyoscyamine (%) in *H. muticus* L hairy root cultures derived from nutrient media supplemented with different concentrations of AgNPs.

AgNPs (mg/L)		Hairy roots			
		Hypocotyl	Leaf	Stem	Root
0	Total alkaloids	2.35 ± 0.05^c^	2.54 ± 0.20^b^	2.39 ± 0.06^c^	3.25 ± 0.18^a^
	Hyoscine	0.008 ± 0.002^bc^	0.011 ± 0.002^b^	0.005 ± 0.004^c^	0.023 ± 0.006^a^
	Hyoscyamine	0.013 ± 0.006^c^	0.02 ± 0.004^b^	0.01 ± 0.002^c^	0.025 ± 0.006^a^
25	Total alkaloids	2.42 ± 0.08^d^	3.17 ± 0.06^b^	2.63 ± 0.03^c^	3.32 ± 0.1^a^
	Hyoscine	0.012 ± 0.004^c^	0.021 ± 0.006^b^	0.018 ± 0.002^b^	0.027 ± 0.005^a^
	Hyoscyamine	0.019 ± 0.006^c^	0.025 ± 0.002^b^	0.021 ± 0.005^bc^	0.032 ± 0.003^a^
50	Total alkaloids	2.63 ± 0.05^d^	3.23 ± 0.09^b^	2.71 ± 0.03^c^	3.45 ± 0.09^a^
	Hyoscine	0.016 ± 0.003^c^	0.025 ± 0.007^b^	0.021 ± 0.003^b^	0.031 ± 0.004^a^
	Hyoscyamine	0.022 ± 0.005^d^	0.032 ± 0.004^b^	0.027 ± 0.003^c^	0.041 ± 0.003^a^
100	Total alkaloids	2.94 ± 0.11^d^	3.42 ± 0.15^b^	3.07 ± 0.09^c^	3.57 ± 0.11^a^
	Hyoscine	0.019 ± 0.003^c^	0.031 ± 0.009^b^	0.023 ± 0.003c	0.037 ± 0.002^a^
	Hyoscyamine	0.027 ± 0.005^c^	0.039 ± 0.003^b^	0.031 ± 0.002^c^	0.046 ± 0.007^a^
200	Total alkaloids	3.15 ± 0.09^d^	3.31 ± 0.05^b^	3.26 ± 0.05^c^	3.43 ± 0.03^a^
	Hyoscine	0.012 ± 0.004^c^	0.027 ± 0.005^a^	0.019 ± 0.003^b^	0.029 ± 0.005^a^
	Hyoscyamine	0.025 ± 0.009^b^	0.035 ± 0.002^a^	0.028 ± 0.003^b^	0.039 ± 0.005^a^

Mean values followed by different letters are signifi^c^antly different according to DMRT at *P* < 0.05.

Means with the same letter are not significantly different, and different letters show significant differences (P <0.05).

### Antimicrobial activity

**Figure 4:**
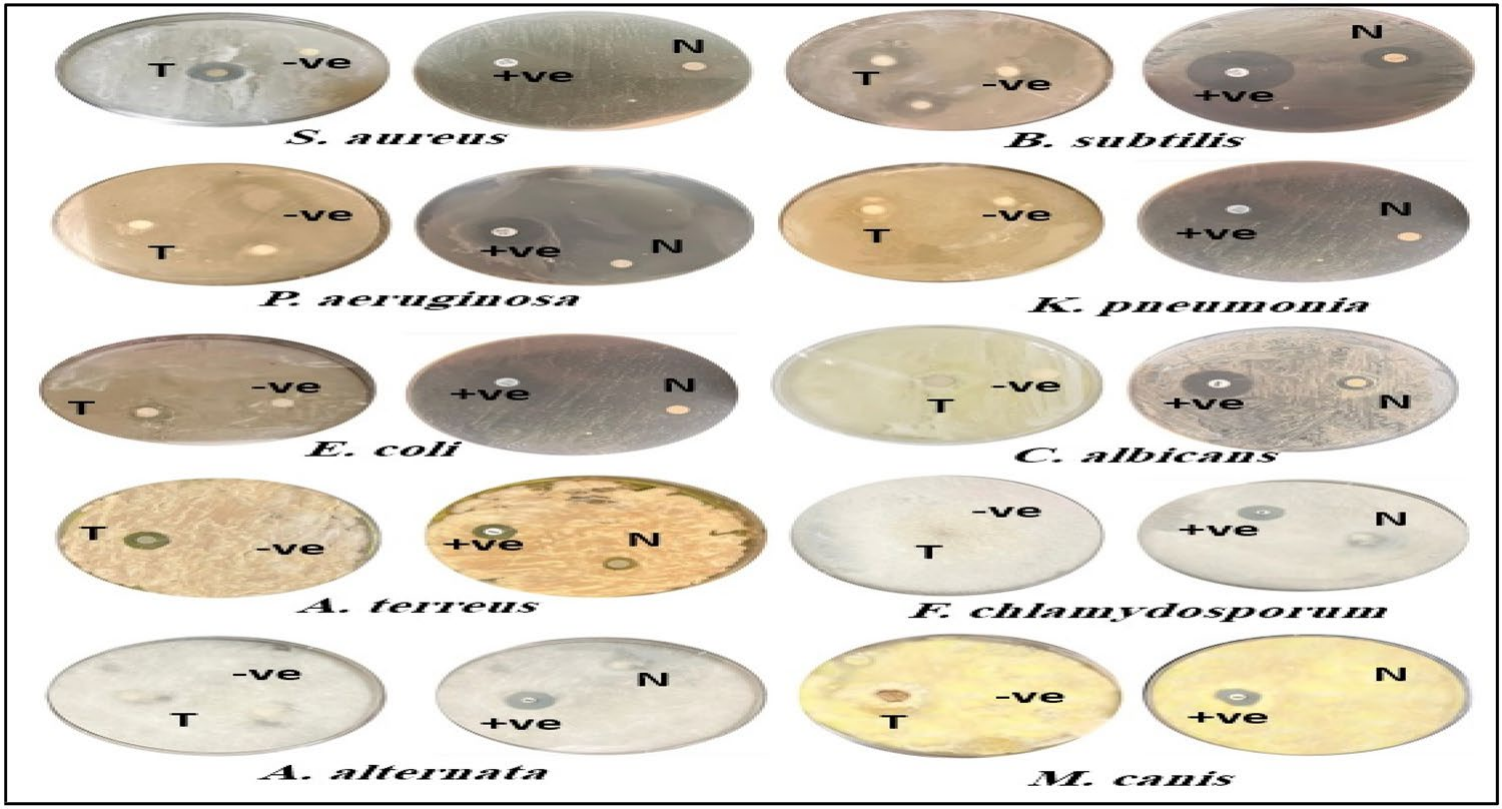
Antimicrobial activity of the ethanolic extract of Transgenic hairy roots (T) and normal plant (N) from hypocotyl explants of *Hyoscyamus muticus* L in comparison with positive control (+ ve) and negative control (−ve).

An inhibitory zone assay was used to determine the antimicrobial activity of an ethanolic extract of hypocotyl explants grown in an *H. muticus* L hairy root culture (Table 2 and Fig. 4). Ethanolic extract inhibited the growth of several bacterial and fungal strains tested. The inhibitory diameter values range from 9.0 to 20.3 (mm) among different bacterial species. The ethanolic extract exhibited strong antibacterial activity against *B. subtilis* (NBRC: 13,719) and *S. aureus* (ATCC 29,213), but only had a weak effect against *E. coli* (ATTC11775). However, there was no observable effect against *P. aeruginosa* (ATTC35639) and *K. pneumoniae* (ATTC35639). In yeast tests, the ethanolic extract suppresses the development of *Candida albicans*. Furthermore, it was effective against *Aspergillus terreus* (SQu14026) and *Microsporum canis* (IFM 45,829) but ineffective against *Fusarium chlamydosporum* (F25) and *Alternaria alternata*.

**Table 2:** Antimicrobial activity of the ethanolic extract of hairy roots and normal plant from hypocotyl explants of *Hyoscyamus muticus* L. as decided by the disc diffusion method.

Microorganisms	Inhibition zones (mm)			
	Transgenic hairy roots extract	Normal plant Extract	Positive control	Negative control
*S. aureus* (ATCC 29,213)	18.2 ± 0.37	9.3 ± 0.47	20.0 ± 0.81	−ve
*B. subtilis* (NBRC:13,719)	20.3 ± 0.72	15.6 ± 1.69	25.0 ± 0.81	−ve
*P. aeruginosa* (ATTC35639)	−ve	−ve	17.0 ± 2.1	−ve
*E. coli* (ATTC11775)	9.0 ± 0.83	7.0 ± 0.81	19.0 ± 0.81	−ve
*K. pneumonia* (ATCC 15,380)	−ve	−ve	22.3 ± 1.6	−ve
*C. albicans* (ATTC90028)	19.5 ± 0.91	9.3 ± 0.47	19.0 ± 0.81	−ve
*A. terreus* (NIH2624)	14.2 ± 0.80	6.6 ± 0.47	14.6 ± 0.47	−ve
*F. chlamydosporum* (F25)	−ve	−ve	16.3 ± 0.47	−ve
*A. alternata* (Te19)	−ve	−ve	17.0 ± 0.81	−ve
*M. canis* (IFM 45,829)	8.5 ± 1.20	−ve	16.0 ± 3.7	−ve

( ±), Standard deviation; (−ve), negative result; (Ampicillin), positive control for bacterial strains and (Fluconazole), for fungal strains; (Dimethyl sulfoxide), negative control.

llnn.

## Discussion

Defense Secondary metabolites are activated by biological or non-biological elicitors. In hairy root cultures, *Agrobacterium rhizogenesis,* as a biological elicitor, stimulates alkaloid synthesis in *H. muticus*, including hyoscine and hyoscyamine. Tepfer^32^ demonstrated that *Agrobacterium* may transfer large plasmids carrying T-DNA into the genome of host plant cells. Many species with hairy rootstocks have been propagated using *Agrobacterium rhizogenes*. Plants generated from different rootstock cultures have piqued the interest of researchers due to their genetic and metabolic stability, high growth rates, and ability to generate by-products in proportions comparable to wild type roots.

Nanoparticles as nano-biological elicitors have emerged as novel plant biosynthesis triggers in recent years^33,34^. Due to their chemical and physical properties, NPs with a high surface-to-volume ratio, the capacity to influence electron exchange, and strong surface contact capabilities are potential candidates for modifying the redox state and impacting plant growth, performance, and quality^35^.

Application of Ag-NPs increased artemisinin by 3.9-fold in 20-day-old hairy root cultures of *Artemisia annua* L.^36^ Hydroponically grown *Bacopa monnieri* L. treated with copper-based NPs (Cu) boosted saponin, alkaloid, flavonoid, and phenol content, as well as antioxidant activity^37^. Celastrol, a therapeutic phytochemical^38^, was enhanced after AgNPs were applied to adventitious and hairy root cultures of *Celastrus paniculatus*. Recently, cell suspension cultures of *Hypericum perforatum* L. treated with metal (Ag, Au, Cu, Pd) and metal oxide (CeO_2_, CuO, TiO_2_, ZnO) NPs enhanced the production of a variety of bioactive secondary metabolites^39^.

*Agrobacterium rhizogenes*-mediated technique is effective in inducing *H. muticus* hairy roots and secondary metabolic products. Two *Agrobacterium* strains were examined for their potential to stimulate *rhizogenesis* in our study, and A4 was shown to be the most virulent, resulting in the rapid formation of hairy roots at the fastest growth rates. The most common strain, 15,834, demonstrated great virulence only in the production of neoplastic callus but not in the propagation of infection via rhizomes.

These results are in agreement with the study conducted by Vanhala et al.^40^, who observed that the *Agrobacterium* strains (LBA9402, A4, 15,834, and C58CI) had an impact on the growth rate, maturation, and synthesis of hyoscyamine in transformed *H. muticus*. Specifically, the C58C1 strain resulted in the highest level of alkaloids, indicating its effectiveness. Similarly, Nicoll et al.^41^ found that, the 15,834 strain was the least effective in promoting the development of hairy roots in *H. muticus*, which aligns with our findings. Additionally, Yoshimatsu and Shimamura^42^ reported that *A. niger* infection in opium poppy hypocotyls reduced its production. They noted that unlike the 15,834 strain that produced hairy roots, *A. rhizogenes* MAFF 03–01,724 and an unknown strain induced Calli.

Williams and Ellis^43^ reported that 15,834 strain was the most potent and efficient in promoting the development of hair roots in *Catharanthus roseus* G. Don, as shown by Brillanceau et al.^44^. Additionally, Vanhala et al.^40^ found that the *Agrobacterium* strains had an impact on the maturation, expansion, and hyoscyamine production of *H. muticus* mutant root cultures.

The enhanced growth and yield of *Avena sativa* resulting from nanoparticle treatment may be attributed to the enhancement of essential element concentrations in plant tissues, as well as the elevation of peroxidase, catalase, and nitrite reductase activities in such tissues^45^.

Furthermore, AgNPs have been found to affect the production of total alkaloids, including hyoscyamine and hyoscine, in *H. muticus*. Shakeran et al.^46^ reported that nano-silver treatment caused a 2.4-fold increase in atropine concentration in hairy root cultures of *D. metel* after elicitation, which is consistent with our findings. Sharafi et al.^11^ also observed that NPs play a crucial role in elevating artemisinin production in *A. annua*. *H. reticulatus* treated with SiO_2_ NPs 100 and 200 (mg/L) for 24 h accumulated the most total phenolic and flavonoids contents as reported by Ahad et al*.*^47^. Also, HPLC analysis showed that after 24 h of treatment with 100 (mg/L) SiO_2_ NPs, *H. reticulatus* transformed roots accumulated much more hyoscyamine 140.15 (mg/g Fresh Weight) and scopolamine 67.71 (mg g^−1^ Fresh Weight) than non-treated roots, by 1212% and 272%, respectively. Moreover, optimal doses and exposure times were found to increase hyoscyamine and scopolamine synthesis in *H. reticulatus* hairy root cultures treated with iron oxide NPs^40^. The results also showed that hyoscyamine and scopolamine concentrations in the treated hairy root cultures were higher than in the control group.

Elicitation of *H. muticus* hairy root culture with sliver nanoparticles might be due to the activation of enzymatic reactions involved in the synthesis pathways of tropane alkaloids. Sliver nanoparticles are a novel elicitor of the hairy root culture of *H. muticus*.

The ethanolic extract derived from the hairy root culture of *H. muticus* L. exhibited potent antibacterial activity against *B. subtilis* (NBRC:13,719) and *S. aureus* (ATCC 29,213), but it only displayed weak activity against *E. coli* (ATTC11775), and it did not show any activity against *P. aeruginosa* (ATTC35639) and *K. pneumoniae* (ATTC35639). However, the ethanolic extract demonstrated strong anti-*Candida albicans* (*C. albicans*) activity in yeast tests. Additionally, it was highly effective against *A. terreus* (SQu14026) and *M. canis* (IFM 45,829), but it had no effect against *F. chlamydiosporum* (F25) and *A. alternata*.

According to Almalki^48^, the methanol extract of *H. muticus* hairy root cultures demonstrated mild antimicrobial activity against various bacteria, including *B. subtilis* (MTCC441), *E. faecalis* (ATCC 29,212), *S. aureus* (ATCC 25,923), *S. epidermidis* (MTCC 3615), *E. coli* (ATCC25923), *K. pneumoniae* (ATCC 15,380), and *P. aeroginosa* (ATCC 27,853). However, it did not exhibit any activity against *A. niger* but was effective against several other fungi. In contrast, Elsharkawy et al.^49^ found that the methanolic extract of *H. muticus* aerial parts was successful in reducing the growth of *S. aureus* and *Bacillus cereus*. They also observed an inhibitory effect on *E. coli*, *K. pneumoniae*, and *P. aeruginosa*. However, Kebaili et al.^50^ demonstrated that the *H. muticus* hydroalcoholic extract was effective against *Escherichia coli* and *Staphylococcus aureus* but not against *Pseudomonas aeruginosa.* The antimicrobial activity of a methanol extract of *H. muticus* leaves showed mild effectiveness against *Escherichia coli, Pseudomonas aeruginosa*, and *Listeria monocytogenes*, and weak activity against *Staphylococcus aureus,* while showing no effectiveness against fungal growth^51^**.**

## Conclusion

The results revealed that *Agrobacterium* strain A4 was more effective in promoting hairy root formation compared to strain 15,834. However, the highest dry weight of hairy roots was obtained from hypocotyl explants, and hairy roots derived from root explants showed significant accumulation and production of secondary metabolites such as total tropane alkaloids, hyoscine, and hyoscyamine. Moreover, treatment with AgNPs at 100 mg/L resulted in the highest production of total tropane alkaloids, including hyoscine and hyoscyamine, in hairy roots obtained from hypocotyl explants. Additionally, the ethanol extracts of hairy roots obtained from hypocotyl explants exhibited high to mild antimicrobial activity against tested gram-positive bacteria, gram-negative bacteria, and fungi. These findings demonstrate that nano-silver can be a promising elicitor for enhancing the production of secondary metabolites in *Hyoscyamus muticus* hairy root cultures, which has potential economic benefits.

## Supplementary Information

Supplementary Information.

## Data Availability

The data in this study are available upon request from the corresponding author.
