# Supplementary material for: Effect of silver nanoparticles on tropane alkaloid production of transgenic hairy root cultures of Hyoscyamus muticus L. and their antimicrobial activity
Source: Sci Rep. 2023 Jun 27;13:10397. doi: 10.1038/s41598-023-36198-x (PMC10300048; doi:10.1038/s41598-023-36198-x)
Supplement: Supplementary file 1 — Supplementary Information. [file 41598_2023_36198_MOESM1_ESM.pdf]

Effect of silver nanoparticles on Tropane alkaloid production of transgenic hairy root cultures of *Hyoscyamus muticus* L and their antimicrobial activity

Aisha M. Abdelkawy[1], Shifaa O. Alshammari[2], Hebat-Allah A. Hussein[1,3], Inas M.M. Abou El-Enain[1], Eman S. Abdelkhalek[1], Asmaa M. Radwan*[1], Sahar K.M. Kenawy[1], Doaa A.M. Maaty[1], Nermine N. Abed[1], Sabry S. H[1] and Abeer Mohsen[4]

Polymerase chain reaction analysis

Total DNA was isolated using a DNA isolation kit from both converted and nontransformed roots (Fermentas, Germany). Using primers specific to the rol B gene, a PCR analysis was conducted. 5'-tggatcccaaattgctattccacga3' and 5'-ttaggcttctttcttcaggtttactgcagc-3' were the primers designed to amplify rol B. In a final volume of 20 L of 1 PCR buffer, the PCR reactions comprised 3 mM MgCl2, 1 mM of each dNTP (Fermentas Co.), 0.4 M of each particular primer, 1 U of Taq DNA polymerase (Fermentas Co.), and 20 ng genomic DNA or 10 ng pRi plasmid DNA used as a positive control. The PCR settings were 94°C (5 min), 30 cycles of three stages [94°C (1 min), 58°C (1 min), and 72°C (30 s)], followed by an extension at 72°C (10 min). Electrophoresis on 1% agarose under UV trans-illuminator showed the PCR products.

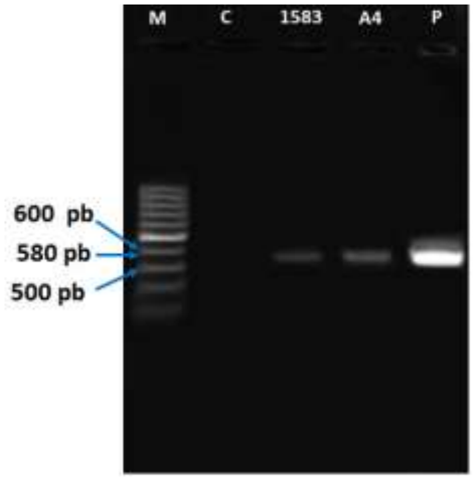

Fig. (S1). PCR analysis for detection of T-DNA in normal roots (C) and hairy roots of Hyoscyamus muticus L treated with *Agrobacterium* strain 1583 andA4, M: Molecular weight marker, (P) plasmid DNA from *Agrobacterium rhizogenes* (positive control); rol B (580 bp)
